# Supplementary material for: Prediction of Primary Tumor Sites in Spinal Metastases Using a ResNet-50 Convolutional Neural Network Based on MRI
Source: Cancers (Basel). 2023 May 30;15(11):2974. doi: 10.3390/cancers15112974 (PMC10252134; doi:10.3390/cancers15112974)
Supplement: Supplementary file 1 [file cancers-15-02974-s001.zip › cancers-2383331-supplementary.pdf]

Supplemental Materials

Table S1: MRI-Sequence Parameters of the Imaging Protocol

	FOV (cm^2)	Slice thickness (mm)	Slice gap (mm)	TR/TE (ms)
Cervical vertebra				
SAG T2 FRFSE	28×28	3.0	0.5	2700/120
SAG T1 FSE	28×28	3.0	0.5	710/8.0
SAG T2 IDEAL	28×28	3.0	0.5	2500/85
Thoracic vertebra				
SAG T2 FSE	36×36	3.0	0.5	2700/120
SAG T1 FSE	36×36	3.0	0.5	700/9.0
SAG T2 IDEAL	36×36	3.0	0.5	2500/85
Lumbar and sacral vertebra				
SAG T2 FSE	30×30	4.0	0.5	3100/120
SAG T1 FSE	30×30	4.0	0.5	700/10
SAG T2 FS	30×30	4.0	0.5	3300/85

Note.—MRI = magnetic resonance imaging. FOV = field of view. TR = rime to repeat. TE = time to echo. SAG = sagittal. FRFSE = fast relaxation fast spin echo. FSE = fast spin echo. IDEAL = iterative decomposition of water and fat with echo asymmetry and least-squares estimation. FS = fat suppression.

Table S2: The numbers of images in the training set and testing set.

	T1WS	T2WS	T2WS-FS	Overall
Training set	3571	3557	3537	10665
Testing set	395	393	392	1180

Note.—T2WS = T2-weighted sequence. T1WS = T1-weighted sequence. T2WS-FS = fat-suppressed T2 sequence.

Table S3: Architecture of the ResNet-50.

ResNet-50 (w/o head)	Conv1	7 * 7, s2
	max pooling, stride 2	
	Conv2	$\begin{bmatrix} 1 * 1, & 64 \\ 3 * 3, & 64 \\ 1 * 1, & 256 \end{bmatrix} \times 3$
	Conv3	$\begin{bmatrix} 1 * 1, & 128, s2 \\ 3 * 3, & 128 \\ 1 * 1, & 512 \end{bmatrix} \& \begin{bmatrix} 1 * 1, & 128 \\ 3 * 3, & 128 \\ 1 * 1, & 512 \end{bmatrix} \times 3$
	Conv_4	$\begin{bmatrix} 1 * 1, & 256, s2 \\ 3 * 3, & 256 \\ 1 * 1, & 1024 \end{bmatrix} \& \begin{bmatrix} 1 * 1, & 256 \\ 3 * 3, & 256 \\ 1 * 1, & 1024 \end{bmatrix} \times 5$
	Conv_5	$\begin{bmatrix} 1 * 1, & 512, s2 \\ 3 * 3, & 512 \\ 1 * 1, & 2048 \end{bmatrix} \& \begin{bmatrix} 1 * 1, & 512 \\ 3 * 3, & 512 \\ 1 * 1, & 2048 \end{bmatrix} \times 2$

Note.—'s2' represents the layer has stride 2. Skip connections and fully-connected layers are not included in the table for clarity.

Gender and Age Classifier

Our research has tried amounts of standard machine learning methods including naïve bayes, support vector classifier (SVC), k-nearest neighbors (kNN), random forest and logistic regression (LR) to analyses the gender and age information. Specifically, for naïve bayes, we use gaussian naïve bayes to fit the vectorized features to do a 5-class classification. For support vector classifier, we use standard support vector classifier with 3-degree RBF kernel. The squared regularization penalty is 1.0 and the gamma is set as the reciprocal of the number of patients in the training dataset. For kNN, we set k equals 3. All neighbors have the same weight on the distance. For random forest, we search the hyperparameter by grid search. The optimal number of estimators is 10. For LR, we limit the max iteration to 5000. The implementation of these methods is based on sci-kit learn toolkits (https://scikit-learn.org/stable/index.html). Data preparation is done with Pandas (https://pandas.pydata.org/).
